# Re-examination of the risk of dementia after dengue virus infection: A population-based cohort study

**DOI:** 10.1371/journal.pntd.0011788

**Published:** 2023-12-06

**Authors:** Yu-Wen Chien, Hsin-I Shih, Yu-Ping Wang, Chia-Yu Chi

**Affiliations:** 1 Department of Public Health, College of Medicine, National Cheng Kung University, Tainan, Taiwan; 2 Department of Occupational and Environmental Medicine, National Cheng Kung University Hospital, College of Medicine, National Cheng Kung University, Tainan, Taiwan; 3 Department of Emergency Medicine, National Cheng Kung University Hospital, College of Medicine, National Cheng Kung University, Tainan, Taiwan; 4 School of Medicine, College of Medicine, National Cheng Kung University, Tainan, Taiwan; 5 National Mosquito-Borne Diseases Control Research Center, National Health Research Institutes, Miaoli County, Taiwan; 6 National Institute of Infectious Diseases and Vaccinology, National Health Research Institutes, Miaoli County, Taiwan; 7 Department of Microbiology & Immunology, College of Medicine, National Cheng Kung University, Tainan, Taiwan; McGill University Faculty of Medicine and Health Sciences, CANADA

## Abstract

Dengue infection can affect the central nervous system and cause various neurological complications. Previous studies also suggest dengue was associated with a significantly increased long-term risk of dementia. A population-based cohort study was conducted using national health databases in Taiwan and included 37,928 laboratory-confirmed dengue patients aged ≥ 45 years between 2002 and 2015, along with 151,712 matched nondengue individuals. Subdistribution hazard regression models showed a slightly increased risk of Alzheimer’s disease, and unspecified dementia, non-vascular dementia, and overall dementia in dengue patients than the nondengue group, adjusted for age, sex, area of residence, urbanization level, income, comorbidities, and all-cause clinical visits within one year before the index date. After considering multiple comparisons using Bonferroni correction, only overall dementia and non-vascular dementia remained statistically significant (adjusted SHR 1.13, 95% CI 1.05–1.21, p = 0.0009; E-value 1.51, 95% CI 1.28-NA). Sensitivity analyses in which dementia cases occurring in the first three or five years after the index dates were excluded revealed no association between dengue and dementia. In conclusion, this study found dengue patients had a slightly increased risk of non-vascular dementia and total dementia than those without dengue. However, the small corresponding E-values and sensitivity analyses suggest the association between dengue and dementia may not be causal.

## Author summary

Dengue fever is a mosquito-borne disease caused by the dengue virus. Dengue infection has been shown to affect the central nervous system and cause various complications during the acute stage. Two previous studies suggested that dengue virus had a long-term effect on the risk of developing dementia, but there were limitations in disease diagnosis in the studies. Our study found that dengue was associated with a slightly increased risk of dementia. However, the small corresponding E values suggest that the association between dengue and dementia was less likely to be causal.

## Introduction

Dengue is caused by the dengue virus (DENV), which has four distinct but closely related serotypes (DENV1–4) and belongs to the family *Flaviviridae*, genus *Flavivirus*. Dengue is transmitted by *Aedes aegypti* and *Aedes albopictus* mosquitoes and has rapidly spread worldwide in recent decades, likely due to a combination of factors, including global warming, unplanned urbanization, and increased international travel and trade [1]. It has been estimated that about 390 million DENV infections occur per year, of which 96 million manifest clinically with a broad spectrum of manifestations ranging from subclinical disease, mild febrile illness to severe dengue, formerly called dengue hemorrhagic fever or dengue shock syndrome [1,2].

Several flaviviruses, such as Japanese encephalitis virus, West Nile virus, and Zika virus, can infect human nervous system cells and cause neurological damage [3]. Cumulative evidence suggests that DENV infection can also affect the central nervous system and cause various neurological complications, such as encephalitis, encephalopathy, meningitis, stroke, cerebellar syndrome, transverse myelitis, and acute disseminated encephalomyelitis [4,5]. In addition to these neurological disorders during acute infection, two previous studies have investigated whether DENV infection had a long-term effect on the risk of developing dementia using the National Health Insurance Research Database (NHIRD) in Taiwan. The results showed that dengue patients had a significantly higher risk of dementia than people without dengue, with adjusted hazard ratios of 2.23 [95% confidence interval (CI) 1.51–3.53] [6] and 1.71 (95% CI 1.03–2.83) [7], respectively. However, dengue cases in these two studies were identified based on clinical diagnosis, not laboratory-confirmed. Furthermore, these two studies included 398 and 816 dengue cases, respectively, because only a small subset of the NHIRD was used. Recently, the Taiwan’s Centers for Disease Control (Taiwan CDC) released the Notifiable Disease Dataset of Confirmed Cases (NDDCC) to be linked with the NHIRD. We obtained the list of laboratory-confirmed dengue patients from the dataset and found that the accuracy of dengue diagnosis in the previous two studies was only 46.0% and 64.1%, respectively.

Considering the rising incidence of dengue and the aging population globally, it is essential to investigate the association between dengue and dementia to better estimate the disease burden caused by DENV. It can be challenging to select uninfected individuals for comparison in countries where dengue fever is prevalent, and most people have been infected multiple times [8]. Nonetheless, in Taiwan, with its low incidence of dengue and availability of comprehensive national databases, we have an exceptional opportunity to conduct cohort studies with long followup. Therefore, the objective of this study was to include all laboratory-confirmed dengue cases diagnosed between 2002 and 2015 and the entire NHIRD in Taiwan to re-examine the risk of dementia after DENV infection.

## Materials and methods

### Ethics statement

This study was approved by the Institutional Review Board of National Cheng Kung University Hospital (B-ER-106-184) and National Health Research Institutes (EC1100107-E). All the data were deidentified, and thus, the requirement for informed consent was waived.

### Data source and study population

The single-player National Health Insurance (NHI) Program was launched in 1995 and currently covers 99.9% of over 23 million population in Taiwan [9]. The NHIRD, derived from the claim data from this program and maintained by the Health and Welfare Data Science Center, has been widely used for academic research. Encrypted identification numbers are used to protect personal information, and the database can be linked to dozens of databases from Taiwan’s public health and welfare system for academic research purposes.

Newly laboratory-confirmed dengue cases between 2002 and 2015 were identified using the NDDCC database. The laboratory criteria for confirmed cases during the study period included: 1) isolation of DENV; 2) detection of DENV nucleic acid by real-time reverse transcription-quantitative polymerase chain reaction (RT-qPCR); 3) detection of high-titer anti-DENV IgM or IgG using capture ELISAs in the acute stage up until 2009; 4) a four-fold or greater increase in IgG titer between paired acute- and convalescent-phase serum samples [10]; 5) positive nonstructural protein 1 (NS1) testing using the DENV NS1 Ag strip rapid test kit [11,12]. Patients who did not have valid ID numbers, gender information, or birth year information were excluded. In addition, we set the date of symptom onset as the index date for each dengue case, and all of the included subjects must fulfill the following criteria: 1) aged 45 or above [6]; 2) without any diagnosis of dementia before the index date; 3) surviving for more than 30 days from the index date. For each dengue patient, four nondengue control subjects were randomly selected from all enrollees of the NHI Program in Taiwan and individually matched based on age, sex, area of residence, and the index date after excluding those with missing information or a diagnosis of dengue. The matched nondengue subjects shared the same index date as their corresponding dengue patients, and individuals with a dementia diagnosis prior to the index date or those who passed away within 30 days following the index date were excluded.

### Study outcome and follow-up

In this study, dementia was classified into three types: Alzheimer’s disease, vascular disease, and unspecified dementia, as previously done by Chu *et al*. [6]. Firstly, the identification of Alzheimer’s disease relied on not only the specific ICD-9-CM code of 331.0 and ICD-10-CM code of G30 but also other dementia ICD-9/10-CM codes with the prescription of medications, including cholinesterase inhibitors and memantine (S1 Table). Then vascular dementia was identified in patients without Alzheimer’s disease by the specific ICD-9-CM code of 290.4 and ICD-10-CM code of F01. After excluding Alzheimer’s disease and vascular dementia, subjects with the other ICD-9/10-CM codes listed in the y Table were defined as patients with unspecified dementia. Dementia cases were defined by at least two outpatient visits or one hospital admission with the relevant ICD-9/10-CM codes or medication documented by board-certified neurologists or psychiatrists during the follow-up period. Furthermore, given that vascular dementia is more closely associated with comorbidities such as hypertension, hyperlipidemia, and diabetes, and it has a distinct pathogenesis from Alzheimer’s disease and unspecified dementia, we have grouped the latter two as non-vascular dementia. All subjects in the study cohort were followed up from the index dates to the end of 2018 unless the study outcome or death occurred first.

### Covariates

The personal characteristics and socioeconomic variables considered in this study included age, sex, area of residence, level of urbanization [13] and monthly income. The Charlson Comorbidity Index (CCI) [14] and diseases potentially affecting mental health, including cerebrovascular diseases, traumatic brain injury, hypertension, dyslipidemia, diabetes mellitus, depressive disorder, alcohol use disorder, substance use disorder, were also considered as potential confounders to assess comorbid conditions of the dengue cases and nondengue subjects, based on relevant ICD-9-CM codes from at least three outpatient visits or one hospital admission before the index dates. Additionally, the all-cause outpatient visits within the previous year of the index dates were calculated to control potential detection bias due to healthcare usage.

### Statistical analysis

The comparisons between the dengue and nondengue groups were performed using the standardized mean difference (SMD), and an SMD < 0.1 indicated a negligible difference between groups [15]. The incidence rates of total dementia and different dementia types were calculated as the number of new events during follow-up divided by the total follow-up time after DENV infection in person-years. Kaplan–Meier curves were plotted, along with the log-rank test, to compare the incidence of dementia in the two groups. The association of dementia and dengue was evaluated by the subdistribution hazard ratios (SHRs) and 95% CIs obtained from the Fine-Gray subdistribution hazard models, adjusted for the abovementioned covariates and the competing risk of death. Subgroup analyses stratified by sex were performed to evaluate whether the effect of DENV on dementia differed by sex.

As done by Chu *et al*. [6] to account for the insidious onset of dementia, we performed sensitivity analyses in which dementia cases occurring in the first three or five years after the index date were excluded to reduce the possibility of underdiagnosis of occult dementia at the time of DENV infection All data analyses in this study were performed using SAS 9.4 (SAS Institute, Cary, NC). A post hoc Bonferroni correction was applied to derive an adjusted threshold for p values to correct multiple comparisons for four different outcomes and stratified analyses by sex. For results with statistical significance, the E-value was calculated to assess the robustness of the association to potential unmeasured or uncontrolled confounding [16,17].

## Results

**Fig 1:**
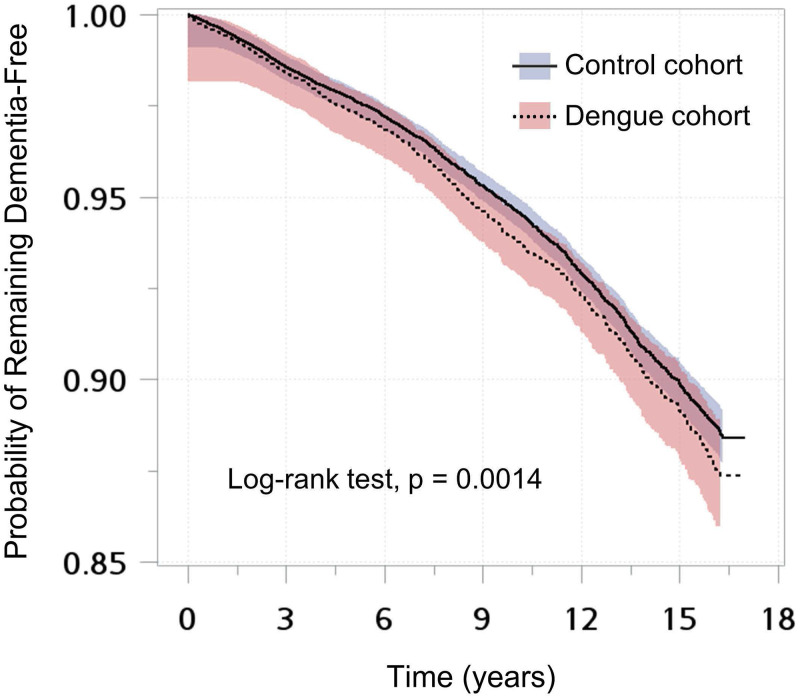
Kaplan-Meier curves with 95% confidence intervals of developing any dementia among patients with dengue and control group.

We identified 37,928 confirmed dengue cases aged ≥ 45 years between 2002 and 2015, along with 151,712 matched nondengue individuals in this study. The mean age of the two groups was 60.71 ± 10.05 years old. The follow-up time in the dengue (median 3.32 years, IQR 3.20–4.26 years) and nondengue groups (median 3.32 years, IQR 3.19–4.25 years) was also similar. The baseline characteristics among dengue and nondengue groups are shown in Table 1. Dengue patients had a higher proportion of dyslipidemia, higher incomes, and urbanized areas than those without dengue, as indicated by SMDs (Table 1). They also had more all-cause clinical visits one year before the index date. Other baseline characteristics and comorbidities did not differ significantly between groups. The Kaplan-Meier curves showed a higher risk of dementia among dengue patients than the control group (log-rank test p = 0.0014, Fig 1).

**Table 1:** Baseline characteristics and incidence of dementia among dengue and nondengue groups.

	Dengue (n = 37928)	Nondengue (n = 151712)	SMD
Age (mean, SD)	60.71 (10.05)	60.71 (10.05)	
Sex (n, %)			
Male	17562 (46.3)	70248 (46.3)	
Female	20366 (53.7)	81464 (53.7)	
Area (n, %)			
Tainan	13453 (35.5)	53812 (35.5)	
Kaohsiung	22823 (60.2)	91292 (60.2)	
Pingtung	850 (2.2)	3400 (2.2)	
Others	802 (2.1)	3208 (2.1)	
Dementia-related comorbidities (n, %)			
Cerebrovascular disease	3816 (10.1)	14859 (9.8)	0.009
Traumatic brain injury	2328 (6.1)	11232 (7.4)	0.049
Hypertension	16873 (44.5)	62990 (41.5)	0.060
Dyslipidemia	14153 (37.3)	49476 (32.6)	0.100*
Diabetes mellitus	8562 (22.6)	32114 (21.2)	0.034
Depressive disorder	3620 (9.5)	12279 (8.1)	0.052
Alcohol use disorder	214 (0.6)	1012 (0.7)	0.013
Substance use disorder	609 (1.6)	2490 (1.6)	0.003
CCI score (mean, SD)	1.83 (2.17)	1.71 (2.18)	0.057
Urbanization level (n, %)			
Urban	29471 (77.7)	84613 (55.8)	0.460*
Suburban	8053 (21.2)	55633 (36.7)	0.326*
Rural	404 (1.1)	11466 (7.6)	0.249*
Income (n, %)			
Not-employed	7339 (19.4)	30023 (19.8)	0.011
Low	13598 (35.6)	63935 (42.1)	0.128*
High	16991 (44.8)	57754 (38.1)	0.138*
Incidence of any dementia (n, %)	1132 (3.0)	4002 (2.6)	0.021
Age at diagnosis of any dementia (mean, SD)	76.58 (7.62)	76.45 (7.74)	
Duration between enrollment and dementia (years, SD)	4.66 (4.33)	4.70 (4.29)	
Dementia type (n, %)			
Alzheimer’s disease	311 (0.8)	1046 (0.7)	0.015
Vascular dementia	139 (0.4)	509 (0.3)	0.005
Unspecified dementia	682 (1.8)	2447 (1.6)	0.015
All-cause clinical visits one year before index date (mean, SD)	21.93 (19.19)	19.90 (19.18)	0.106*

SD: standard deviation; SMD: standardaried mean difference; CCI: Charlson Comorbidity Index

* Indicating SMD > 0.1

The results of the subdistribution hazard models, which compare the risk of dementia between the dengue and nondengue groups, are presented in Table 2. To account for multiple testing, the Bonferroni post hoc method required a significance level of p<0.0033 (4 different outcomes and stratified analyses by sex; total 15 tests in Table 2). Subdistribution hazard models showed a slightly increased risk of Alzheimer’s disease, unspecified dementia, non-vascular dementia, and overall dementia in dengue patients than the nondengue group, adjusted for age, sex, area of residence, urbanization level, income, comorbidities, CCI score, and all-cause clinical visits within one year before the index date. After considering multiple comparisons, only non-vascular dementia (adjusted SHR 1.13, 95% CI 1.05–1.22, p = 0.0010, Table 2) and overall dementia (adjusted SHR 1.13, 95% CI 1.05–1.21, p = 0.0009) remained statistically significant. The corresponding E-values were 1.51 (95% CI 1.28-NA) and 1.51 (95% CI 1.28-NA), respectively. Stratified analyses by sex showed that only male dengue patients had a significantly increased risk of non-vascular dementia (adjusted SHR 1.22, 95% CI 1.09–1.36, p = 0.0004; E-value 1.74, 95% CI 1.40-NA) and overall dementia (adjusted SHR 1.20, 95% CI 1.08–1.33, p = 0.0005; E-value 1.69, 95% CI 1.37-NA). No significant association between DENV infection and dementia was observed in females.

**Table 2:** Risk of developing dementia among dengue patients and nondengue controls.

	Total		Male		Female	
	SHR^a^ (95% CI)^b^	p-value^c^	SHR^a^ (95% CI)^b^	p-value^c^	SHR^a^ (95% CI)^b^	p-value^c^
Alzheimer’s disease	1.16 (1.01–1.32)	0.0330	1.31 (1.07–1.62)	0.0102	1.06 (0.89–1.26)	0.4942
Vascular dementia	1.06 (0.87–1.29)	0.5719	1.08 (0.83–1.41)	0.5724	1.03 (0.77–1.36)	0.8653
Unspecified dementia	1.12 (1.02–1.22)	0.0128	1.18 (1.04–1.34)	0.0118	1.08 (0.95–1.22)	0.2379
Non-vascular dementia	1.13 (1.05–1.22)	0.0010*	1.22 (1.09–1.36)	0.0004*	1.07 (0.97–1.19)	0.1791
Overall dementia	1.13 (1.05–1.21)	0.0009*	1.20 (1.08–1.33)	0.0005*	1.07 (0.97–1.18)	0.1670

^a^ Subdistribution hazard ratio adjusted by age, sex, area of residence, urbanization level, income, comorbidities, CCI score, and all-cause clinical visits.

^b^ 95% CIs were not adjusted for multiple comparisons and thus cannot be directly used for hypothesis testing or inference.

^c^ Post hoc adjustment for multiple comparisons by the Bonferroni method required a significance level of p<0.0033.

*Achieved statistical significance after Bonferroni correction for multiple comparisons (p<0.0033).

The results of sensitivity analyses are shown in Table 3. Although four adjusted SHRs in the sensitivity analyses had p-values < 0.05, the smallest p-value was 0.0205, indicating all the SHRs were not statistically significant after considering multiple testing.

**Table 3:** Sensitivity analyses of developing any dementia among patients with dengue and controls.

	Exclusion of observation period			
	> 3 years^a^		> 5 years^b^	
Dengue (Yes vs No)	SHR^c^	p-value	SHR^c^	p-value
All dengue				
Alzheimer’s disease	1.09 (0.90–1.32)	0.3892	0.93 (0.75–1.17)	0.5572
Vascular dementia	0.98 (0.75–1.29)	0.8875	0.97 (0.71–1.33)	0.8453
Unspecified dementia	1.16 (1.01–1.32)	0.0304	1.11 (0.95–1.30)	0.1935
Non-vascular dementia	1.14 (1.02–1.27)	0.0205	1.05 (0.92–1.20)	0.4449
Overall dementia	1.12 (1.01–1.24)	0.0314	1.04 (0.92–1.18)	0.4931
Male dengue				
Alzheimer’s disease	1.24 (0.92–1.67)	0.1535	0.98 (0.69–1.40)	0.9181
Vascular dementia	0.85 (0.57–1.27)	0.4283	0.84 (0.52–1.35)	0.4639
Unspecified dementia	1.15 (0.94–1.40)	0.1670	1.01 (0.79–1.28)	0.9675
Non-vascular dementia	1.18 (1.00–1.39)	0.0469	1.00 (0.82–1.22)	0.9801
Overall dementia	1.12 (0.96–1.31)	0.1375	0.97 (0.81–1.17)	0.7554
Female dengue				
Alzheimer’s disease	1.00 (0.78–1.29)	0.9741	0.91 (0.68–1.22)	0.5234
Vascular dementia	1.13 (0.78–1.64)	0.5254	1.10 (0.72–1.70)	0.6514
Unspecified dementia	1.17 (0.98–1.40)	0.0841	1.20 (0.97–1.48)	0.0941
Non-vascular dementia	1.12 (0.96–1.29)	0.1481	1.09 (0.92–1.30)	0.3224
Overall dementia	1.12 (0.98–1.29)	0.0999	1.10 (0.94–1.29)	0.2442

^**a**^ 179487 dengue patients and 717948 nondengue subjects

^**b**^ 38235 dengue patients and 152940 nondengue subjects

^**c**^ Subdistribution hazard ratio adjusted by age, sex, area of residence, urbanization level, income, comorbidities, CCI score, and all-cause clinical visits.

## Discussion

Our study found slightly increased risks of non-vascular and overall dementia in dengue patients, particularly in males. However, these associations were not observed in the sensitivity analyses, which excluded dementia cases occurring in the first few years of follow-up to avoid under-diagnosis of occult dementia when and shortly after dengue was diagnosed. In addition, the corresponding E-values and their lower confidence limits were not very large, implying that the evidence of causality was very weak because little unmeasured confounding could explain away the observed associations [16,17]. Hence, our study did not provide robust evidence to support a causal link between DENV infection and dementia. Our findings contrast with two previous studies that reported more substantial association between dengue and dementia (HRs of 2.23 and 1.71), where the associations remained statistically significant in sensitivity analyses [6,7]. Several factors could account for these discrepancies. Firstly, the dengue patients in the previous studies were diagnosed based on clinical presentation, rather than laboratory confirmation, because DENV NS1 rapid tests were not widely available before 2014 [18]. Secondly, these studies comprised of only 398 and 816 dengue cases, respectively, since they utilized only a subset of the NHIRD. In contrast, our study incorporated the entirety of the NHIRD and included a total of 37,928 dengue cases. Finally, we applied a post hoc Bonferroni correction to adjust the threshold for p values to correct for multiple comparisons, a step that was not undertaken in the previous studies.

Dengue is considered an acute viral disease. The majority of the patients develop subclinical or mild symptoms. However, in some cases, the disease can progress to severe dengue, which may lead to complications such as severe bleeding, organ impairment, or plasma leakage [1]. Neurological manifestations have been increasingly reported in dengue patients, with prevalence rates ranging from 0.5 to 21% [4,19]. The neuropathogenesis of DENV can be classified into three categories: (1) metabolic disturbance causing encephalopathy; (2) direct viral invasion causing encephalitis, meningitis, myositis, and myelitis; and (3) autoimmune reactions causing diseases such as acute disseminated encephalomyelitis and Guillain-Barré syndrome [6,20]. The two most common neurological complications caused by DENV are encephalopathy and encephalitis [4,6,21]. DENV-induced encephalopathy may present as decreased sensitivity, cognitive dysfunction, seizures, and personality disorders, including sudden mania, depression, anxiety, psychosis, and agoraphobia [4]. Dengue encephalopathy usually occurs in patients with severe dengue, with a mortality rate as high as 46.7% [22]. Nevertheless, mild encephalopathy with rapid onset but reversible cognitive impairment has been reported in dengue patients [23]. Although DENV encephalitis can be mild, with reversible functional deficiencies, it can also result in severe structural damage with corresponding functional defects and sequelae [3,24–30]. We further examined whether patients diagnosed with severe dengue (defined in S2 Table) exhibited a higher risk of subsequent dementia. However, our results indicated that severe dengue was not associated with an increased risk of developing dementia (S3 Table).

Dementia currently ranks as the seventh leading cause of death globally and is one of the major causes of disability and dependency among the elderly population [31]. Dementia is an acquired disorder characterized by a significant decline from one’s previous level of cognition involving one or more cognitive domains (learning and memory, language, executive function, complex attention, perceptual-motor, and social cognition) [32]. It should be viewed as a syndrome that develops over time, with various underlying causes, rather than as one particular disease [33]. Cognitive impairment and dementia have been linked to several neurotropic viruses, such as cytomegalovirus, human alphaherpesvirus 1, and alphaherpesvirus 2, probably through neuroinflammation after infection [34–36]. Increased risk of dementia has also been reported in survivors of West Nile virus infection [37]. DENV was considered non-neurotropic before; however, growing evidence of neurotropism and neuro-invasion in dengue has gradually changed this view because DENV can cause various neurological complications during the acute stage [4]. Our study reveals a marginally increased risk of non-vascular dementia and overall dementia among patients with dengue; however, further research is needed to determine whether this association is indeed causal.

Our study has several strengths. First, this population-based cohort study had a large sample size of laboratory-confirmed outpatient and inpatient dengue cases. Second, national medical claims data in Taiwan have a coverage rate of >99.9% of the whole population, minimizing selection bias caused by loss to follow-up in cohort studies. Third, this study controlled many potential confounders, including comorbidities, demographics, and socioeconomic factors. Fourth, the frequency of all-cause clinical visits was adjusted to account for surveillance bias. Fifth, two sensitivity analyses were performed. Sixth, E-values were used to access the evidence of causality in this observational study [16,17]. Finally, our analyses took into account multiple testing by employing the Bonferroni method, thereby reducing the likelihood of false-positive findings. While the Bonferroni correction can sometimes be overly conservative, potentially leading to an increased rate of false negatives [38], we have also applied the Holm-Bonferroni method, which provided consistent results.

This study also has some limitations. One major limitation was that the diagnosis of dementia primarily relies on claim data from the NHI Program rather than a standardized assessment of all individuals in the study group. The possibility of missing codes or coding errors might lead to misclassification. In addition, dementia patients who did not seek medical help could not be detected in the NHIRD and might therefore be misclassified. However, we tried to improve the accuracy of dementia diagnosis by using a stricter definition, only ICD codes by neurologists and psychiatrists. We also adjust all-cause clinical visits to minimize potential detection bias. Another limitation was that some nondengue subjects might be misclassified because DENV infections could be asymptomatic or cause mild symptoms and thus be unrecognized or underreported. However, this misclassification bias should be slight since dengue is currently not endemic in Taiwan; therefore, outbreaks are usually concentrated in some hot spots, and the overall incidence and seroprevalence remain low [39–41]. In addition, one of the criteria for laboratory confirmation of dengue involved detecting DENV IgM or IgG in a single serum sample prior to 2009. This method may have resulted in some degree of misclassification due to cross-reactivity with other flaviviruses. Since dengue is not endemic in Taiwan and the seroprevalence was low before 2009, the misclassification bias is unlikely to be significant. However, the Notifiable Disease Dataset of Confirmed Cases, our primary data source, lacks detailed information on the specific laboratory tests employed for diagnosing dengue patients, making it impossible to calculate the proportions of participants diagnosed by each test. Furthermore, with a median follow-up time of only 3.32 years, the sensitivity analyses that excluded dementia cases occurring within the first three or five years of follow-up had a smaller sample size, leading to decreased statistical power. Finally, some risk factors for dementia, such as smoking habits, alcohol use, body weight, daily activity, diet, educational level, and family history, were unavailable in NHIRD.

## Conclusion

In conclusion, this study found dengue patients only had a slightly increased risk of non-vascular dementia and total dementia than those without dengue. However, the small corresponding E values and sensitivity analyses suggest the association between dengue and dementia may not be causal. Additional research is required to definitively establish whether or not there is a causal relationship between dengue infection and dementia.

## Supporting information

S1 Table List of ICD codes for identifying diseases in this study. (DOCX)

S2 Table Definition of severe dengue. (DOCX)

S3 Table Risk of developing dementia among patients with severe dengue and controls. (DOCX)
