# Supplementary material for: Re-examination of the risk of dementia after dengue virus infection: A population-based cohort study
Source: PLoS Negl Trop Dis. 2023 Dec 6;17(12):e0011788. doi: 10.1371/journal.pntd.0011788 (PMC10699621; doi:10.1371/journal.pntd.0011788)
Supplement: S1 Table — (DOCX) [file pntd.0011788.s001.docx]

S1 Table. List of ICD codes for identifying diseases in this study.

|  | ICD-9-CM | |
| --- | --- | --- |
| Dementia (for excluding patients with dementia before index dates) | 290, 294.1, 294.2, 331.0, 331.1, 331.2, 331.82 | |
|  |  | |
| Dementia type (for identifying outcomes) | ICD-9-CM (until 2015) | ICD-10-CM (since 2016) |
| Alzheimer’s disease |  | |
| medications not-required | 331.0 | G30 |
| medications required | 290.0, 290.1, 290.2, 290.3, 290.4, 294.1, 294.2, 331.1, 331.2, 331.82 | F01, F02, F03, G31.0, G31.1, G31.83 |
| Vascular dementia | 290.4 | F01 |
| Unspecified dementia | 290.0, 290.1, 290.2, 290.3, 294.1, 294.2, 331.1, 331.2, 331.82 | F02, F03, G31.0, G31.1, G31.83 |
|  |  |  |
| Comorbidities | ICD-9-CM | |
| Cerebrovascular disease | 362.34, 430-438 | |
| Traumatic brain injury | 800, 801, 803, 804, 850-854, 959.01 | |
| Hypertension | 401-405 | |
| Dyslipidemia | 272 | |
| Diabetes mellitus | 250 | |
| Depressive disorder | 296.2, 296.3, 300.4, 311 | |
| Alcohol use disorder | 291, 303, 305.0 | |
| Substance use disorder | 292, 304, 305.1-305.9 | |
