# Supplementary material for: Re-examination of the risk of dementia after dengue virus infection: A population-based cohort study
Source: PLoS Negl Trop Dis. 2023 Dec 6;17(12):e0011788. doi: 10.1371/journal.pntd.0011788 (PMC10699621; doi:10.1371/journal.pntd.0011788)
Supplement: S2 Table — (DOCX) [file pntd.0011788.s002.docx]

S2 Table. Definition of severe dengue.

| 1. Death reported in the Notifiable Disease Dataset of Confirmed Cases (NDDCC) released by the Taiwan CDC. |
| --- |
| 1. Hospitalized within two weeks after dengue symptom onset with one of the following: 2. admitted to an intensive care unit 3. use of ventilator 4. use of inotropes or vasopressors 5. having the following ICD-9 codes: dengue hemorrhagic fever (065.4), pleural effusion (511.9), ascites (789.59), acute respiratory failure (518.81), gastrointestinal bleeding (530.82, 531.0, 531.2, 532.0, 532.2, 533.0, 533.2, 534.0, 534.2, 535.01, 578) |
