# Supplementary material for: Re-examination of the risk of dementia after dengue virus infection: A population-based cohort study
Source: PLoS Negl Trop Dis. 2023 Dec 6;17(12):e0011788. doi: 10.1371/journal.pntd.0011788 (PMC10699621; doi:10.1371/journal.pntd.0011788)
Supplement: S3 Table — (DOCX) [file pntd.0011788.s003.docx]

S3 Table. Risk of developing dementia among patients with severe dengue and controls.

| N=13045 | Total |  | Male |  | Female |  |
| --- | --- | --- | --- | --- | --- | --- |
|  | SHR^a^ (95% CI)^b^ | p-value | SHR^a^ (95% CI)^b^ | p-value | SHR^a^ (95% CI)^b^ | p-value |
| Alzheimer’s disease | 0.99 (0.69-1.42) | 0.9528 | 1.33 (0.78-2.25) | 0.2949 | 0.78 (0.46-1.31) | 0.3477 |
| Vascular dementia | 0.89 (0.50-1.58) | 0.6804 | 1.19 (0.49-2.90) | 0.6990 | 0.79 (0.36-1.72) | 0.5465 |
| Unspecified dementia | 1.12 (0.86-1.45) | 0.4209 | 1.03 (0.72-1.48) | 0.8794 | 1.23 (0.85-1.78) | 0.2682 |
| Non-vascular dementia | 1.08 (0.87-1.33) | 0.4970 | 1.12 (0.83-1.51) | 0.4645 | 1.06 (0.79-1.44) | 0.6974 |
| Overall dementia | 1.05 (0.86-1.28) | 0.6278 | 1.12 (0.85-1.49) | 0.4218 | 1.02 (0.77-1.35) | 0.8913 |

^a^ Subdistribution hazard ratio adjusted by age, sex, area of residence, urbanization level, income, comorbidities, CCI score, and all-cause clinical visits.

^b^ 95% CIs were not adjusted for multiple comparisons and thus cannot be directly used for hypothesis testing or inference.
